# Micro-robotic percutaneous targeting of type II endoleaks in the angio-suite

**DOI:** 10.1007/s11548-024-03195-y

**Published:** 2024-05-29

**Authors:** Gerlig Widmann, Johannes Deeg, Andreas Frech, Josef Klocker, Gudrun Feuchtner, Martin Freund

**Affiliations:** 1 Department of Radiology, Medical University of Innsbruck, Anichstr. 35, 6020 Innsbruck, Austria; 2 Department of Vascular Surgery, Medical University of Innsbruck, Innsbruck, Austria

**Keywords:** Robotics, Navigation, Interventional radiology, Accuracy, Angiography, Abdominal aortic aneurysm, Endoleak

## Abstract

**Purpose:**

Endovascular aneurysm repair has emerged as the standard therapy for abdominal aortic aneurysms. In 9–30% of cases, retrograde filling of the aneurysm sac through patent branch arteries may result in persistence of blood flow outside the graft and within the aneurysm sac. This condition is called an endoleak type II, which may be treated by catheter-based embolization in case of continued sac enlargement. If an endovascular access is not possible, percutaneous targeting of the perfused nidus remains the only option. However, this can be very challenging due to the difficult access and deep puncture with risk of organ perforation and bleeding. Innovative targeting techniques such as robotics may provide a promising option for safe and successful targeting.

**Methods:**

In nine consecutive patients, percutaneous embolization of type II endoleaks was performed using a table-mounted micro-robotic targeting platform. The needle path from the skin entry to the perfused nidus was planned based on the C-arm CT image data in the angio-suite. Entry point and path angle were aligned using the joystick-operated micro-robotic system under fluoroscopic control, and the coaxial needle was introduced until the target point within the perfused nidus was reached.

**Results:**

All punctures were successful, and there were no puncture-related complications. The pre-operative C-arm CT was executed in 11–15 s, and pathway planning required 2–3 min. The robotic setup and sterile draping were performed in 1–2 min, and the alignment to the surgical plan took no longer than 30 s.

**Conclusion:**

Due to the small size, the micro-robotic platform seamlessly integrated into the routine clinical workflow in the angio-suite. It offered significant benefits to the planning and safe execution of double-angulated deeply localized targets, such as type II endoleaks.

## Introduction

Abdominal aortic aneurysms are characterized by localized dilatation of the abdominal aorta of more than 3 cm [1]. They occur in about 1.4% of adults between the ages of 50 and 84 in the U.S. and represent about 75% of all aortic aneurysms. The most common complication is an aneurysm rupture, which may lead to potentially fatal internal bleeding. Over the last years, endovascular aneurysm repair (EVAR) has emerged as the standard therapy for abdominal aortic aneurysms at a sac diameter of ≥ 5.5 cm for men and ≥ 5.0 cm for women, with high success rates. Nevertheless, various types of endoleaks resulting in persistence of blood flow outside the graft and within the aneurysm sac may occur preventing its complete thrombosis [2]. Retrograde filling of the aneurysm sac through patent branch arteries is the most common type of endoleak. This is defined as “type II endoleak” and is estimated to appear in approximately 9–30% of abdominal EVAR patients [3]. Although spontaneous resolution of type II endoleaks is possible (ranges from 33 to 50% within the first year), sac enlargement of more than 5 mm may require treatment via transcatheter therapeutic embolization of the feeding arteries and/or the endoleak nidus [4, 5]. Access to the aneurysm sac is typically achieved using an endovascular transarterial approach selectively targeting the feeding vessels via inferior mesenteric artery, accessory renal, median sacral, or internal iliac arteries. In cases where a transarterial approach is not possible, the only alternative access is the direct percutaneous puncture of the perfused endoleak nidus under imaging guidance [6]. This procedure can be very demanding due to difficult access and deep puncture with risk of organ perforation and bleeding. Application of a robotic system in the angio-suite may provide a useful option for safe and successful targeting of the aneurysm sac.

## Material and methods

In our tertiary institution, between 01.01.2020 and 01.01.2023, nine patients emerged with a persistent and growing type II endoleak after endovascular aneurysm repair of an infrarenal abdominal aortic aneurysm. The indication for treatment was decided based on a multidisciplinary team discussion consisting of vascular surgeons and interventional radiologists. In all patients, treatment via endovascular route was not possible, and therefore, the percutaneous approach was the only option. Based on the high experience of our institution in stereotactic navigated procedures, we decided for use of an interventional micro-robotic system to guide the percutaneous puncture of the aneurysm sac in the angio-suite. Informed consent was obtained from all patients.

Before treatment, all patients received routine contrast-enhanced computed tomography (CT) or magnetic resonance imaging (MRI) of their aneurysm to obtain high-resolution images in native, arterial, and delayed phases for exact localization of the perfused nidus of the type II endoleak and pre-selection of the puncture route via prone or supine position.

On the day of the treatment, all patients were prepared for general anesthesia in the angio-suite. All except one were positioned prone for targeting the endoleak. Patients were draped, and the access area was prepared. The angio-suite consisted of a single-plane C-arm angiography system with a flat-panel detector (Allura Xper FD10, Philips Medical Systems, Best, The Netherlands). A rotational C-arm CT scan was performed to acquire the pre-procedural imaging for planning using the XperGuide planning tool. This tool required definition of a target point and an entry point. The target point was set in the center of the perfused endoleak nidus. To help localize the target point in the non-contrast-enhanced C-arm CT scan, the images were closely correlated to the previous contrast-enhanced CT or MRI images. The entry point was defined on the skin surface to ensure a safe access path from the skin to the target point without harm to critical structures such as bowel, ureter, vena cava, and other vessels. For this purpose, two lateral trajectory views and a probe view were available.

**Fig. 1:**
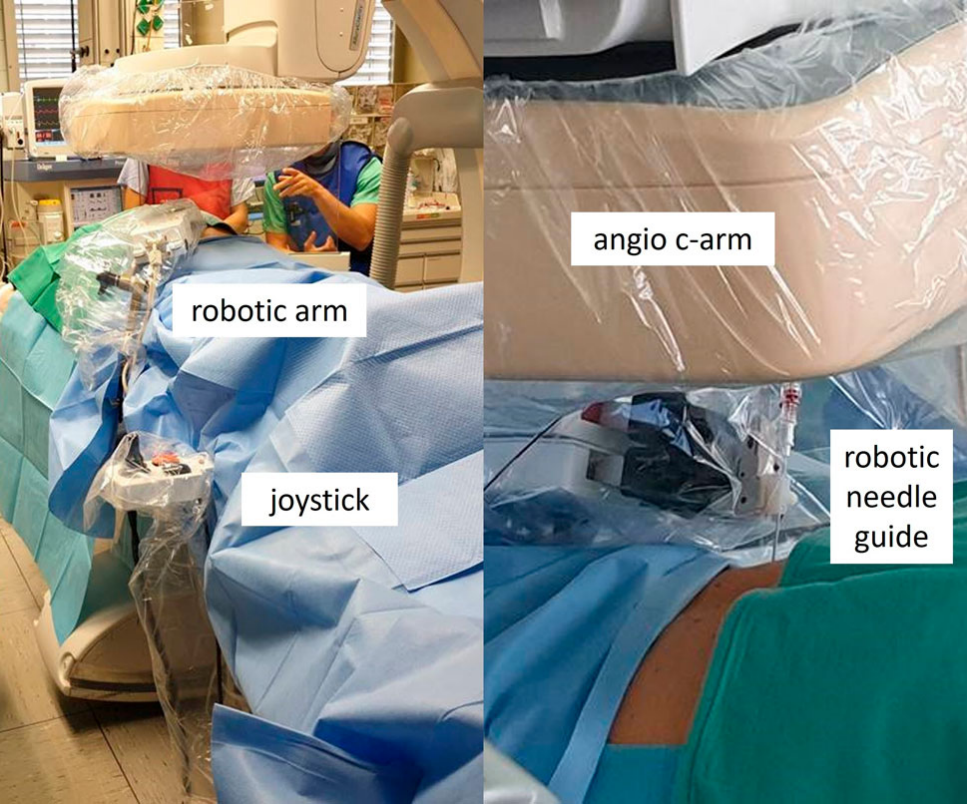
Micro-robotic setup in the angio-suite

**Fig. 2:**
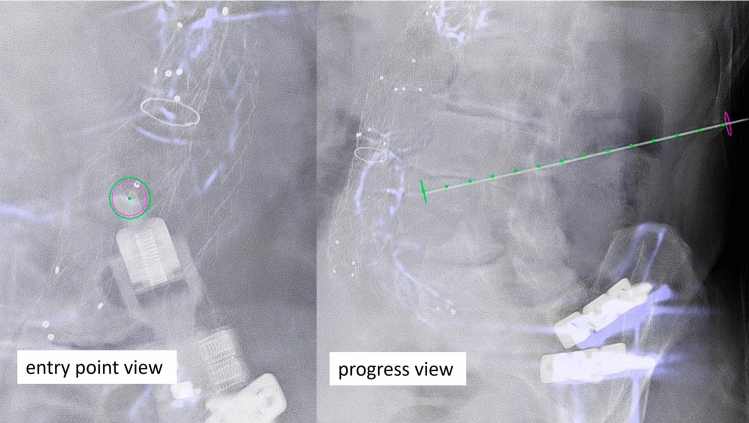
Targeting documentation using “Entry Point View” to align entry and path angles, and “Progress View” to check the penetration depth to the target point

A micro-robotic, arm-based targeting platform was directly mounted to the angio-table (Interventional Systems, iSYS Medizintechnik GmbH, Kitzbühel, Austria; iSYS1 in the first eight cases and the latest version Micromate in the last case). Based on the information from the XperGuide planning, the C-arm was moved to the “Entry Point View.” This view provided fluoroscopy images exactly onto the planned path. The micro-robotic, arm-based targeting platform was covered with sterile drapes and fixed in close proximity to the entry point (Fig. 1). A blunt fill needle (18 G, 40 mm) was placed in the needle hold of the tip of the targeting platform. Using the telemanipulator joystick, the micro-robotic targeting platform was aligned to both, the entry point and the path angulations under live fluoroscopic control. Perfect alignment was achieved when the operator could look precisely at the path through the hole of the needle (Fig. 2). Then, the C-arm was moved to “Progress View.” This view provided fluoroscopy images at 90 degrees lateral to the planned path and was used to control needle insertion depth. The blunt fill needle was replaced with the interventional sheathed coaxial introducer needle (19 G, 19 cm). This needle was gradually inserted under fluoroscopic control until it reached the exact target point (Fig. 2).

**Fig. 3:**
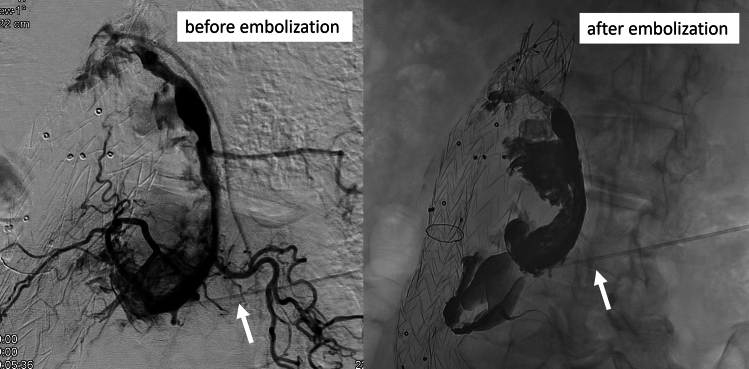
Angiography before embolization showing the extent of the type II endoleak with feeding vessels. Single-shot angiography documents the success of embolization with the opaque liquid embolization material within the type II endoleak and without filling of the feeding vessels

The successful puncturing of the perfused aneurysm sac was indicated via backflow of blood through the coaxial needle. A contrast injector was connected to the coaxial needle, and an angiographic series was performed to visualize the endoleak and potential feeding side branches (see Fig. 3). An Echelon 14 micro-catheter and Fathom 14 micro-wire (Medtronic Trading NL B.V., Eindhoven, The Netherlands) were used to enter the endoleak and to selectively target side branches using conventional fluoroscopic control. If possible, accessible side branches were embolized using micro-coils. The rest of the endoleak was embolized using liquid material (Onyx®, Covidien, Dublin, Republic of Ireland) with the goal to occlude side branches and to minimize inflow into the type II endoleak (Fig. 3).

Contrast-enhanced CT or MR was performed the day after the procedure. The effect of embolization was evaluated by comparing the perfused volume of the endoleak in the pre-treatment images with the perfused post-treatment volume. The volumes were manually segmented using the PACS (picture archiving and communication in medicine) software (Dedalus Healthcare Group AG, Bonn, Germany).

## Results

In this preliminary case series, seven males and two females were treated, with a mean age of 78.2 years, ranging 73–87 years. The micro-robotic-guided puncture to the perfused endoleak nidus was successful in all patients. Excellent alignment of needle to the planned path was observed in the fluoroscopic guidance images (see Fig. 2). There were no puncture-related complications.

Setting up the micro-robotic system on the angio-table and sterile draping took 1–2 min, and aligning it with the surgical path using the telemanipulator joystick took no longer than 30 s (Table 1). Due to the miniature size and arm-based design, no collusion problems occurred during the C-arm positioning in both, “Entry Point View” and “Progress View.” Sufficient access to the patients was warranted. The micro-robotic targeting platform fixed the coaxial introducer needle in place and provided stable access to the perfused endoleak nidus during the entire catheter-based embolization procedure.

**Table 1:** Duration of micro-robotic targeting workflow

Duration of pre-operative C-arm CT	11–15 s
Duration of pathway planning	2–3 min
Duration of draping the robotic system and setup	1–2 min
Duration of robotic alignment	20–30 s

Comparing pre-treatment and post-treatment images, a complete reduction of endoleak perfusion was obtained in five patients, and a significant reduction was documented in the remaining four patients: from 99.8 to 5.2 ml, 64.2 to 2.3 ml, 11.6 to 2.2 ml, and 2.8 to 0.6 ml. Overall perfused volumes before and after embolization are shown in Table 2. During routine follow-up, the aneurysm sac did not progress in size, and the small residual endoleaks remained stable. No re-treatment was indicated for all patients.

**Table 2:** Perfused type II endoleak volume before and after embolization

Perfused volume before treatment ± SD [range] (mL)	24.5 ± 34.1 [2.1–99.8]
Perfused volume after treatment ± SD [range] (mL)	1.1 ± 1.8 [0–5.2]

## Discussion

The micro-robotic percutaneous puncture of the perfused nidus of type II endoleaks was performed with success and without complications. Its miniature size and easy handling compared with other interventional robotic systems allowed the convenient integration of the robotic targeting platform into the routine workflow in the angio-suite [7, 8]. The arm-based targeting platform, which was directly mounted to the angio-table, prevented collision problems when moving the C-arm and enabled easy access to the patient. The time required for setup and sterile draping was 1–2 min, and the duration of the robotic path alignment took no longer than 30 s. Compared with previous robotic systems, the procedure time could be significantly reduced [9].

Percutaneous type II endoleak targeting can be extremely challenging due to the small target size, long path, and narrow corridor for safe access [6]. Selective planning of arbitrary angulated trajectories via target point and entry point definition using different views allowed for selection of the most appropriate path. The micro-robotic needle hold established an accurate needle guidance under fluoroscopic control and provided stability for the subsequent catheter-based embolization. The setup flexibility of the micro-robotic system offered new possibilities to execute trajectories that would typically be rejected using conventional puncture techniques due to the difficulty of maintaining a proper alignment during freehand guidance. The telemanipulator joystick allowed path alignment with minimal fluoroscopic dose. Traditional freehand guidance requires a step-and-go technique that uses repeated C-arm CT scans to document and verify the needle's safe route of access to the target. The applied radiation dose may thus be substantially higher and contribute to increased cumulative effective doses, to both the patient and staff [10]. Furthermore, only the in-plane puncture technique can be used safely because it is extremely important to see the entire needle in the image plane to avoid targeting critical out-of-plane structures. With C-arm CT, oblique out-of-plane trajectories could fundamentally be avoided, even without the use of a robot. However, the possible angulations for a complete rotation of the C-arm to enable true compensation of oblique trajectories are limited. Still, double-angulated or steep-angulated targeting paths may become extremely difficult or even impossible [11].

As an alternative to the robot system, optical navigation systems can be used. These systems are based on a stereoscopic camera, which can localize instruments for navigation based on the patient’s CT data [12]. To facilitate the guidance of tracked instruments, navigation systems may be used in combination with a stereotactic aiming device [13]. These devices work similar to the micro-robotic targeting platform, except the adjustments of target entry and path angulations are set manually using the navigation system. Previous publications have documented improved lateral accuracy using a stereotactic aiming device compared to handheld navigated targeting [14]. Navigation systems can also be operated in the angio-suite and may use C-arm CT image data with similar accuracy to conventional CT [15, 16]. However, using the micro-robotic system in combination with fluoroscopic guidance, an additional stereoscopic camera for optical navigation is not required, and use of fiducial markers or scanning of registration tools for the mandatory image-to-patient registration procedure can be omitted. Nevertheless, the micro-robotic system may also be operated hybridly in combination with optical tracking with excellent puncture accuracy [17]. In terms of clinical applications, the micro-robotic system may be additionally used for a broad spectrum of image-guided interventions, such as stereotactic biopsy of liver, lung, and bone lesions, or stereotactic radiofrequency ablation of liver tumors [18–25].

In conclusion, the micro-robotic platform with easy setup and flexible positioning between the flat panel and the patient seamlessly integrated into the typical percutaneous type II endoleak embolization workflow in the angio-suite. It may offer significant benefits to the procedure execution, providing accurate targeting with remote control under live fluoroscopic imaging, and stable access during the embolization procedure.
