# Correction to: Micro-robotic percutaneous targeting of type II endoleaks in the angio-suite

**DOI:** 10.1007/s11548-024-03271-3

**Published:** 2024-11-07

**Authors:** Gerlig Widmann, Johannes Deeg, Andreas Frech, Josef Klocker, Gudrun Feuchtner, Martin Freund

**Affiliations:** 1 Department of Radiology, Medical University of Innsbruck, Anichstr. 35, 6020 Innsbruck, Austria; 2 Department of Vascular Surgery, Medical University of Innsbruck, Innsbruck, Austria

**Correction to: International Journal of Computer Assisted Radiology and Surgery (2024) 19:1489–1494** 10.1007/s11548-024-03195-y

In the original version of this article, authors’ name Andreas Frech and Josef Klocker were incorrectly written as Frech Andreas and Klocker Josef.

The original article has been corrected.

